# Innovations for effective implementation of guideline-based hypertension care in low-income and middle-income countries

**DOI:** 10.1016/S2214-109X(21)00083-8

**Published:** 2021-03-19

**Authors:** Nitish Naik, Kavita Singh

**Affiliations:** Department of Cardiology, All India Institute of Medical Sciences, New Delhi, India; Centre for Chronic Conditions and Injuries, Public Health Foundation of India, Gurugram, India

Hypertension is the leading preventable cause of death and disability worldwide, accounting for 19·2% (10·8 million) of all deaths globally and 16·4% (2 million) of all deaths in low-income and middle-income countries.^1^ Hypertension is easy to diagnose, treat, and control, yet suboptimal screening and inadequate therapy has resulted in an increased number of patients with uncontrolled disease despite numerous advances over the past two decades. Increasing awareness, treatment, and control of hypertension is predicated on the accessibility and responsiveness of the health system to the needs of its population. Vertically aligned health programmes and a persistent shortage of trained physicians along with their disproportionate concentration in urban as opposed to rural areas have hampered routine periodic screening for non-communicable diseases in most low-income and middle-income countries. Effective hypertension control is thus multifactorial and requires a recalibration of the health system.^2^

The COBRA-BPS study provides useful information on the effectiveness of a complex intervention programme led by community health workers in combating hypertension in rural areas.^3^ In this cluster-randomised trial among 30 communities in Bangladesh, Pakistan, and Sri Lanka, a multicomponent hypertension management programme was evaluated for the budget that is required to scale up the programme and its cost-effectiveness.^4^ The intervention was locally adapted, with targeted education, training of providers, regular blood pressure monitoring and referral, and a financing model to support additional service provision by community health workers. From the health system (public payer) perspective, the investigators found the first-year costs per participant to be US$10·65 for Bangladesh, $10·25 for Pakistan, and $6·42 for Sri Lanka, and recurrent costs per participant ranged from $5·70 (Pakistan) to $6·52 (Bangladesh). Incremental cost-effectiveness ratios ranged from $2270 (in Pakistan) to $4080 (in Sri Lanka) per cardiovascular disease-associated disability-adjusted life-year averted.

Community health workers have traditionally been involved in the delivery of maternal and child health services in most low-income and middle-income countries as well as in HIV/AIDS programmes. Task sharing for hypertension control by community health workers offers a cost-effective way of addressing the community burden of hypertension. A 2019 meta-analysis showed a significant reduction in blood pressure when hypertension care was provided by non-physician health-care workers.^5^ The feasibility and success of a programme delivered by community health workers does pose several potential challenges, however.^6^ The effectiveness of the intervention is highly dependent on many health system factors such as availability, funding, and retention of an appropriately sized team of community health workers; adequate training, supervision, and monitoring of community health workers; the development of context-specific protocols for screening, diagnosing, referring, and treating patients; availability and access to affordable medicines; and clear career pathways for the community health workers. Many community health workers are often already overloaded with multiple responsibilities and are poorly remunerated, leading to poor motivation and job attrition. Designing a programme that is culturally and socially acceptable is a crucial factor that drives the success of the task-sharing strategy; for instance, many community health workers are women, and gender barriers in some communities might impair the effective rollout of a programme led by community health workers.

Compared with the enormous effort spent on research to identify cost-effective treatments in clinical trials, there have been few efforts to integrate new strategies in routine clinical practice. The study by Finkelstein and colleagues^4^ fills an important gap in informing health-care professionals and governments on how to improve hypertension care in rural communities. Progressively more rigorous methods have been used to generate evidence on the cost-effectiveness of interventions for hypertension control. Trial-based cost-effectiveness analyses along with budget impact analyses, as used by Finkelstein and colleagues,^4^ generally provide a more robust estimate of cost-effectiveness than decision-based models.^7^ Despite the remarkably low per-participant cost reported in the trial,^4^ expanding the COBRA programme could require a considerable increase in the health-care budget by at least 20–30% across the three countries. An increase in the out-of-pocket expenditure for pharmacotherapy for hypertension could also affect the willingness to seek and continue care. It is, therefore, important to understand how the COBRA-BPS intervention is valued from the patients, service providers, and societal perspectives in public and private facilities. With the increased implementation of health insurance models by governments in south Asia, further economic data will help to examine how different payers value interventions led by community health workers.

Relative to overall budgets and estimated gains, there are other cost-effective strategies to manage hypertension.^8^ However, despite all the implementation challenges and available cost-effectiveness evidence, task sharing among physicians and health workers should be viewed as an essential and key element for hypertension control in low-income and middle-income countries. The risk is as Toni Morrison said in her epic novel *Song of Solomon*: “If we don’t create the future, the present extends itself”.
